# Supplementary material for: A Raman probe of phonons and electron–phonon interactions in the Weyl semimetal NbIrTe4
Source: Sci Rep. 2021 Apr 14;11:8155. doi: 10.1038/s41598-021-87302-y (PMC8047047; doi:10.1038/s41598-021-87302-y)
Supplement: Supplementary file 1 — Supplementary Information [file 41598_2021_87302_MOESM1_ESM.pdf]

A Raman Probe of Phonons and Electron-phonon Interactions in $NbIrTe_4$

Iraj Abbasian Shojaei [a], Seyyedesadaf Pournia, Congcong Le [b,c], Brenden R. Ortiz [d,e],
Giriraj Jnawali [a], Fu-Chun Zhang [b], Stephen D. Wilson [d,e],
Howard E. Jackson [a], Leigh M. Smith [a],*
[a] *Department of Physics, University of Cincinnati, Cincinnati, OH, USA*
[b] *Kavli Institute of Theoretical Sciences, University of Chinese Academy of Sciences, Beijing 100190, China*
[c]*Max Planck Institute for Chemical Physics of Solids, 01187 Dresden, Germany*
[d] *Materials Department, University of California Santa Barbara, Santa Barbara CA 93106*
[e] *California Nanosystems Institute, University of California Santa Barbara, Santa Barbara CA 93106*

*email: leigh.smith@uc.edu

Supporting information

S1: DFT Calculations of Phonon Modes

The calculations are performed using density functional theory (DFT) as implemented in the Vienna ab initio simulation package (VASP) code.[1]–[3] The Perdew-Burke-Ernzerhof (PBE) exchange-correlation functional and the projector-augmented-wave (PAW) approach are used. Throughout the work, the cutoff energy is set to be 550 eV for expanding the wave functions into plane-wave basis, and the number of k points was set to 4 × 4 × 4 for a 3 × 1 × 1 supercell. The real-space force constants of the supercells were calculated in the density-functional perturbation theory (DFPT)[4] and the phonon frequencies were calculated from the force constants using the PHONOPY code.[5] In our calculations, we adopt the experimental structural parameters (a=3.7903A°, b=12.5207A°, and c=13.1435A°) which have been measured by XRD at the University of California at Santa Barbara by Professor Stephan Wilson’s group.

Fig.1(a) in the main text shows crystal structure of $NbIrTe_4$, and all atoms occupy the same Wyckoff 2a{(0, y, z), (1/2, -y, z+1/2)} with corresponding site symmetry group C_{1h}. According to Characteristics of the table of C_{1h}, the vibrational direction of all $A_{1,2}$ and $B_{1,2}$ phonon modes are limited to “yz” plane or the “x” direction. Fig. S1(a) displays the phonon dispersion of $NbIrTe_4$, and no imaginary frequency is observed throughout the whole Brillouin zone. The density of states (DOS) of $NbIrTe_4$ is shown in Fig. S1(b), where the gray line is total DOS, and red, green and blue lines are the DOS of Nb, Te and Ir atoms, respectively. Range of frequency from 0 to 160 cm^{-1}, the DOS are mainly attributed to the Te atoms, and the peak around 187.202 cm^{-1} is from contributions of Te and Ir atoms.

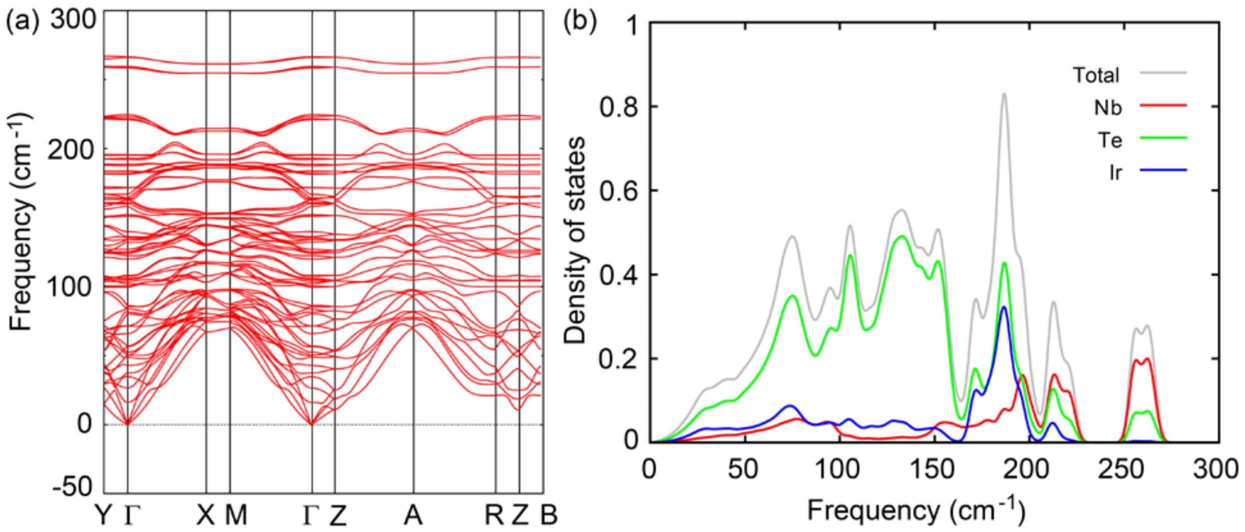

Figure S1: (a) and (b) are the phonon dispersion and DOS of $NbIrTe_4$. Gray is total DOS; Red, green and blue are the DOS of Nb, Te and Ir atoms, respectively.

S2: Angular Dependence of Raman Scattering with |d|/|f|

Fig. S2 shows the angular behavior of three A_1 modes and one A_2 mode for both $e_i \parallel e_s$ and $e_i \perp e_s$ measurements. The left column shows a polar plot of the Raman intensities observed in $e_i \parallel e_s$ measurements and fit using equation (3), while the right column illustrates a polar plot of Raman intensities for $e_i \perp e_s$ measurements and fit to equation (4). The angular dependence of the A_1 modes for $e_i \parallel e_s$ measurements is substantially complex. The ratio of |d|/|f| and φ_{df} have a significant role in determining the *shape* of the intensity as a function of θ (the angle between the laser polarization and the "x" axis of the lab coordinate system). As we see in the left column of the Fig. S2, modes with |d|/|f| > 1 (e.g. the 75.4 cm^{-1} mode) have maxima for the laser polarization aligned with the "x" axis (θ = 0° and 180°), while modes with |d|/|f| < 1 (e.g. the 152.5 cm^{-1} mode) have maxima for the laser polarized along the "y" axis (θ = 90° and 270°). If |d|/|f| = 1 and $\varphi_{df} = 0$, no variation of intensity with θ is observed (the shape is a circle). The 102.3 cm^{-1} mode has |d|/|f| = 1 and $\varphi_{df} = 90°$ which exhibits four-lobed behavior with equal values along both the "x" and "y" directions. The angular dependence of all A_1 modes for the $e_i \perp e_s$ configuration, in contrast, shows simple four-lobed behavior with a maximum intensity aligned with 45° rotated "x" and "y" axes. As is predicted from theory, all A_2 modes display the expected four-lobe pattern as shown in the bottom row of Fig. S2, with the lobes aligned at 45° with respect to the "x" and "y" axes for the $e_i \parallel e_s$ measurements and aligned with the "x" and "y" axes for the $e_i \perp e_s$ configuration. From these observations, it is only possible to extract |d|/|f| for A_1 modes observed in the $e_i \parallel e_s$ configuration.

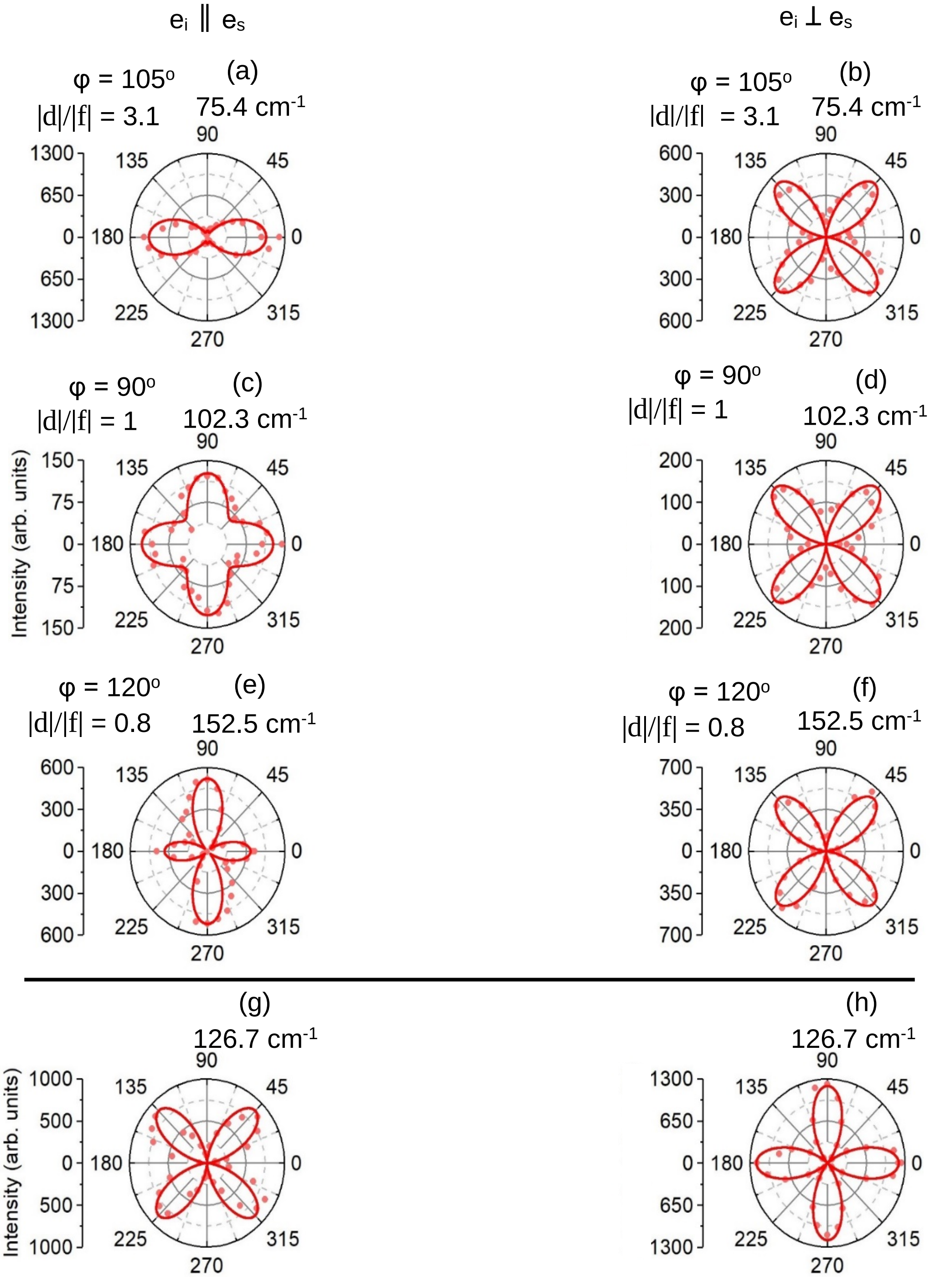

Figure S2: Intensity polar plot of four selected Raman modes of $NbIrTe_4$ as a function of rotation angle which have been measured with 633nm excitation. The first three rows belong to the A_1 irreducible representation and last row belongs to the A_2 irreducible representation. The left column represents a parallel configuration measurement where the solid red curves illustrate the theoretical fitting. The right column is a measurement for a perpendicular configuration with the solid red curves the theoretical fitting.

S3: Angular Dependence of Raman Scattering with ϕ_{df}.

Fig. S3 shows three A_1 modes for both $e_i \parallel e_s$ and $e_i \perp e_s$ measurements. The left column shows a polar plot of Raman scattering intensities measured for $e_i \parallel e_s$ measurements which are fit to equation (3) while the right column illustrates a polar plot of Raman intensities measured for $e_i \perp e_s$ measurements and fit to equation (4). The phonon modes in this figure were chosen in order to illustrate the role of φ_{df} for the angular behavior of A_1 modes for $e_i \parallel e_s$ measurements. Three A_1 modes are shown with similar $|d|/|f| \approx 1.5$ but different φ_{df}'s. One observes no change in the angular dependence (shape) of $e_i \perp e_s$ measurements (right column) by changing φ_{df}, but $e_i \parallel e_s$ measurements (left column) show a distinct variation with φ_{df}. All three modes of the $e_i \parallel e_s$ measurements show approximately two lobed behavior with a maximum intensity along the "x" axis ($\theta = 0^o$ and 180°) because $|d|/|f| >1$. As φ_{df} increases, at $\varphi_{df} = 130^o$, two small lobes appear which are aligned along the "y" axis ($\theta = 90^o$ and 270°). Generally, by increasing φ_{df}, two lobes perpendicular to the initial two lobes grow, and at $\varphi_{df} = 180^o$ it becomes a complete symmetric four lobed plot.

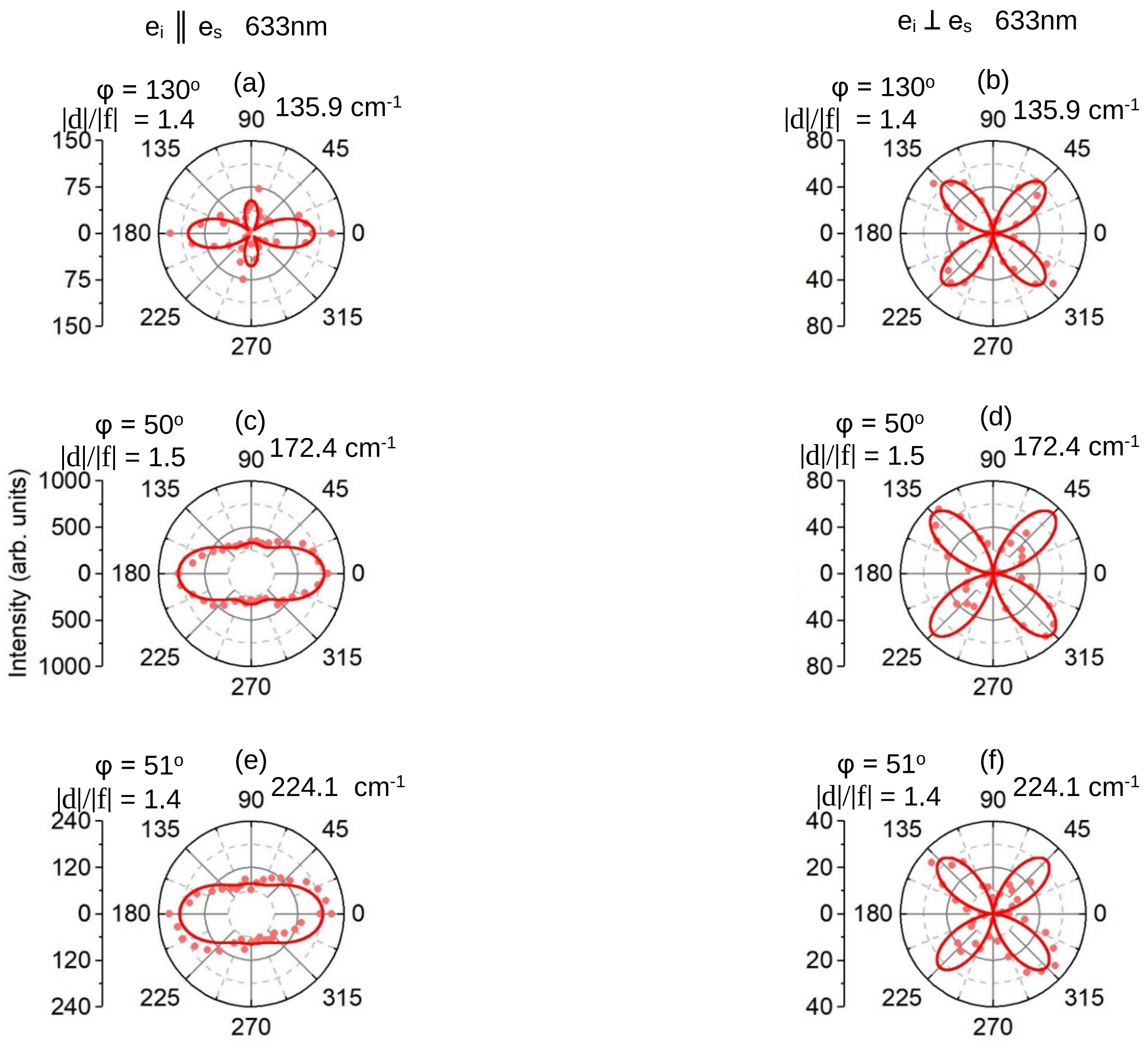

Figure S3: Intensity polar plot of three selected Raman modes of $NbIrTe_4$ with A_1 irreducible representation as a function of rotation angle with almost equal |d|/|f| but different φ_{df} for fitting measurements using 633nm laser excitation. The left column shows parallel configuration measurements where the solid red curves illustrate theoretical fitting. Right column is measurement for perpendicular configuration with the red curves showing the theoretical fitting.

S4: Spectral Calibration

We have used a Ne gas discharge source to calibrate the spectrometer. Fig. S4 shows the Ne spectrum measured by our spectrometer and CCD detector. We have detected 16 Ne emission lines for wavelengths longer than 514 nm to calibrate our Raman measurements when using a 514 nm excitation laser. Figure S5 shows two strong lines of a Ne gas discharge source for wavelengths

longer than 633 nm to calibrate our Raman measurements when using a 633 nm excitation laser. The values of the peaks are shown in Table S1; we compare these values with values which are accepted Ne^{+} lines in literature. The average difference between our measurements and literature values for the Ne lines is 0.018 nm or 0.67 cm^{-1}.

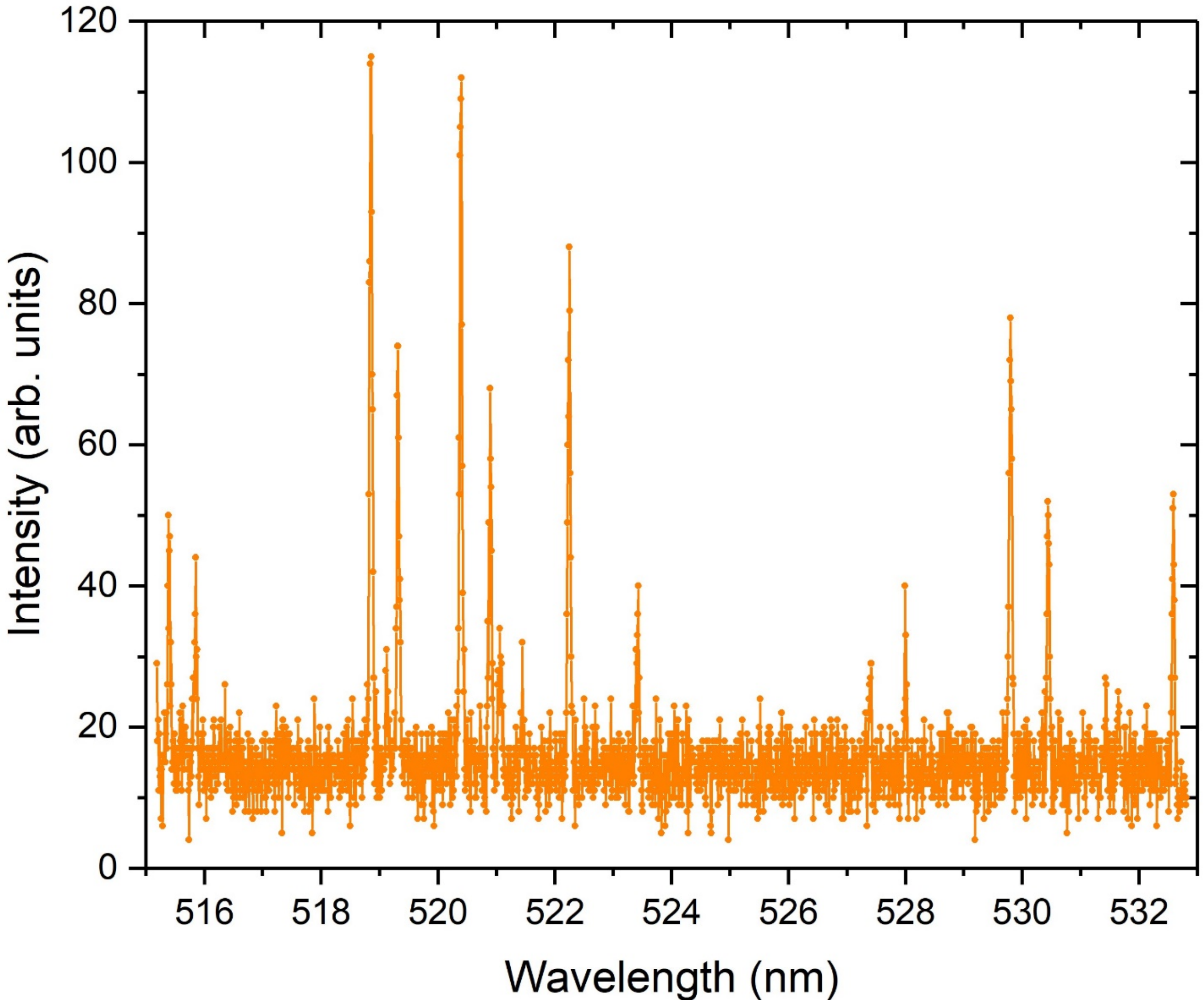

Figure S4: Atomic spectrum of the Ne gas discharge tube from 515 nm to 533 nm.

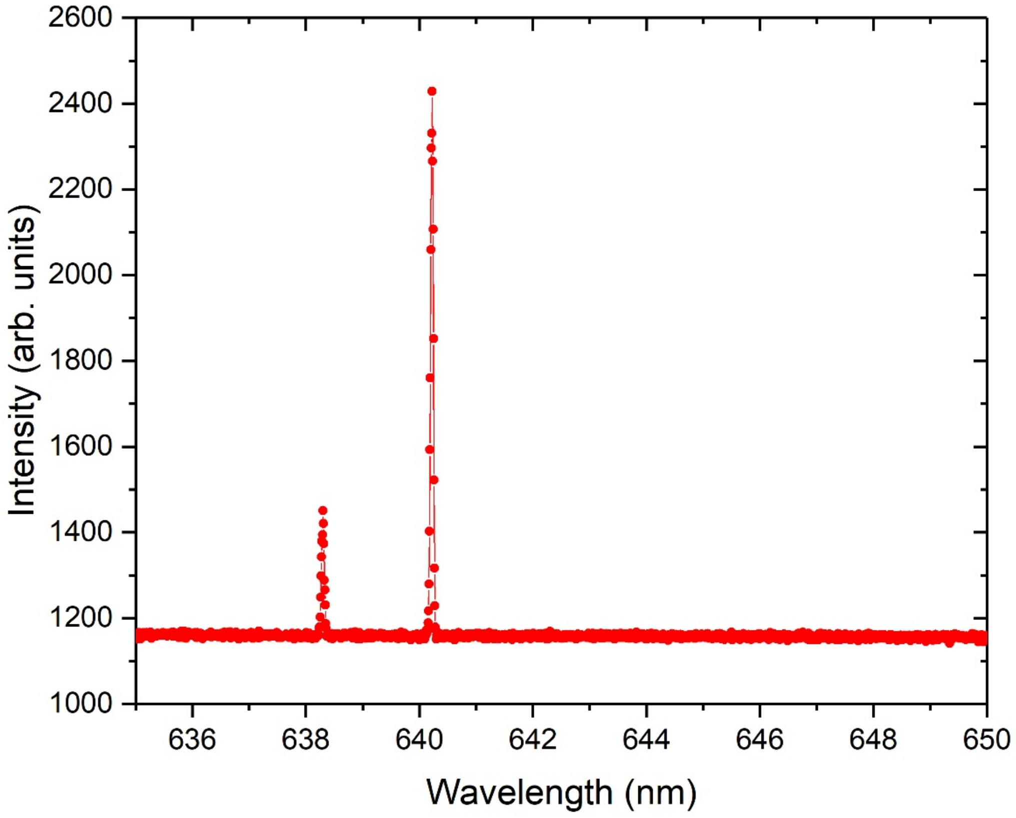

Figure S5: Atomic spectrum of the Ne gas discharge tube from 635 nm to 650 nm.

No	Ne lines in our spectrometer (nm)	Ne lines in literature (nm)	Ne lines width in our spectrometer (cm^{-1})	Measured line – literature line (nm)	Measured line – literature line (cm^{-1})
1	515.41382	515.44271	1.9	-0.02889	1.09
2	515.85339	515.89018	1.6	-0.03679	1.38
3	518.85602	518.86122	1.6	-0.0052	0.19
4	519.12628	519.13223	1.5	-0.00595	0.22
5	519.31799	519.31251	1.6	0.00548	-0.2
6	520.4054	520.38962	1.3	0.01578	-0.58
7	520.89166	520.88648	1.6	0.00518	-0.19
8	521.05646	521.05672	0.5	-0.00026	0.01
9	521.44659	521.43389	1.6	0.0127	-0.47
10	522.25159	522.23517	1.5	0.01642	-0.6
11	523.43439	523.40271	1.5	0.03168	-1.16
12	527.42255	527.40393	0.9	0.01862	-0.67
13	527.99377	528.00853	1.6	-0.01476	0.53
14	529.80414	529.81891	1.4	-0.01477	0.53

15	530.43951	530.47580	1.5	-0.03629	1.29
16	532.59186	532.63960	1.3	-0.04774	1.68
17	638.30054	638.29917	1.1	0.00137	-0.03
18	640.22546	640.2248	1.1	0.00066	-0.02

Table S1: The Ne atomic lines measured with our spectrometer and comparison with literature values

S5: Raman Frequencies of B_1 and B_2 Phonon Modes

Tables S2 and S3 list all Raman active phonon modes of $NbIrTe_4$ with the B_1 and B_2 irreducible representation calculated by DFT. Table S2 shows the energy of 11 B_1 modes and Table S3 shows the energy of 23 B_2 modes.

No	cm^{-1}	No	cm^{-1}	No	cm^{-1}	No	cm^{-1}
1	50.188	4	100.348	7	127.055	10	181.463
2	57.038	5	104.987	8	160.848	11	183.437
3	65.903	6	124.203	9	163.368		

Table S2: Calculated Raman active phonon energy with B_1 symmetry at Γ point by Density Function Theory.

No	cm^{-1}	No	cm^{-1}	No	cm^{-1}	No	cm^{-1}
1	34.719	7	105.950	13	151.020	19	195.510
2	37.384	8	108.060	14	158.093	20	221.688
3	48.814	9	122.498	15	164.224	21	223.131
4	80.062	10	132.031	16	171.826	22	258.347
5	83.788	11	133.861	17	188.621	23	266.455
6	86.271	12	136.206	18	192.993		

Table S3: Calculated Raman active phonon energy with B_2 symmetry at Γ point by Density Function Theory.

S6: Normal Modes and Angular Dependence for all observed Phonon modes

Fig. S6 shows the normal modes and angular dependence for all A_1 and A_2 phonon modes detected in our experiment. Results for both 514 and 633 nm excitation are shown for A_1 modes detected in the $e_i \parallel e_s$ configuration.

A_1

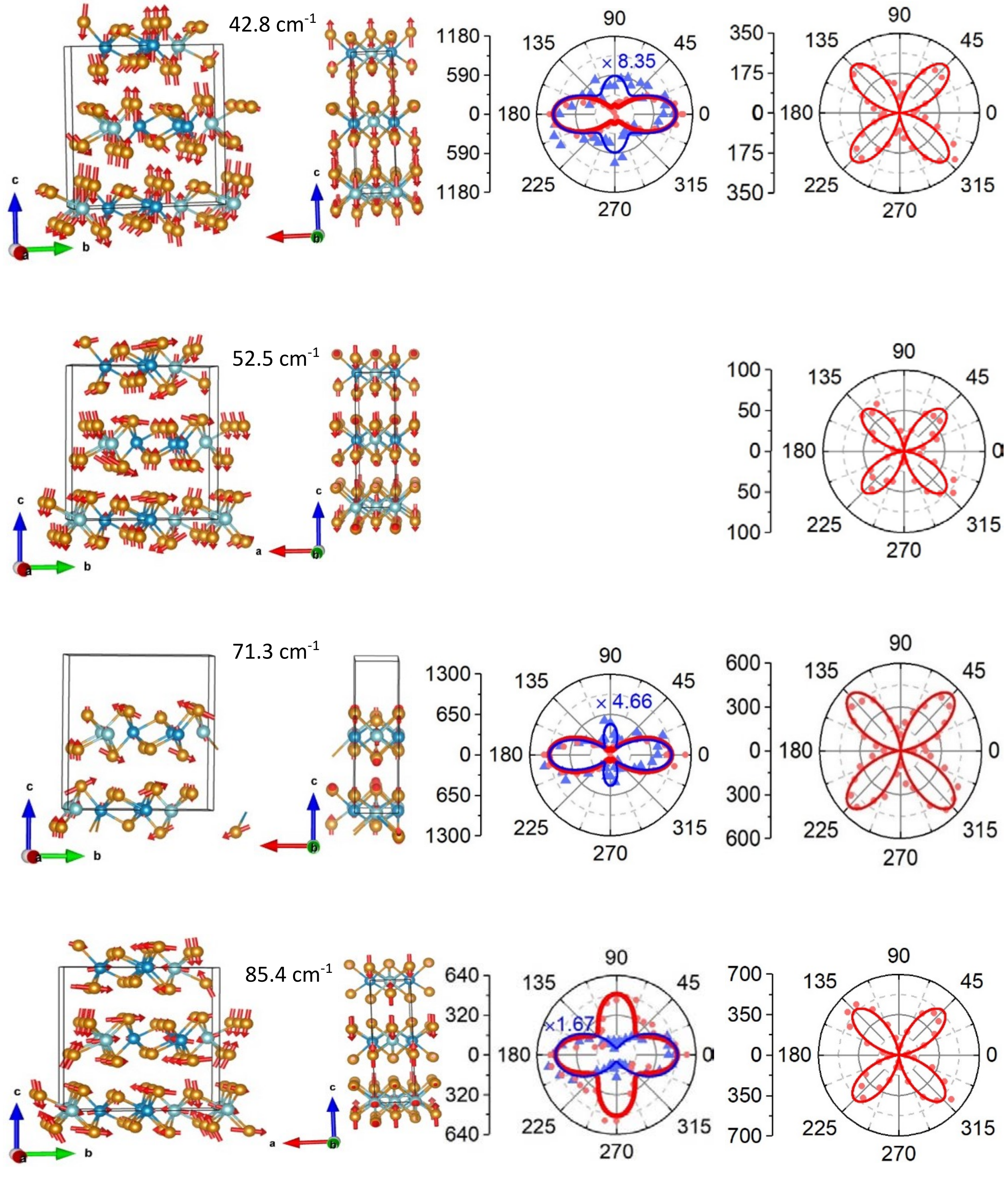

42.8 cm-1
52.5 cm-1
71.3 cm-1
85.4 cm-1
× 8.35
× 4.66
×1.67
a
b
c

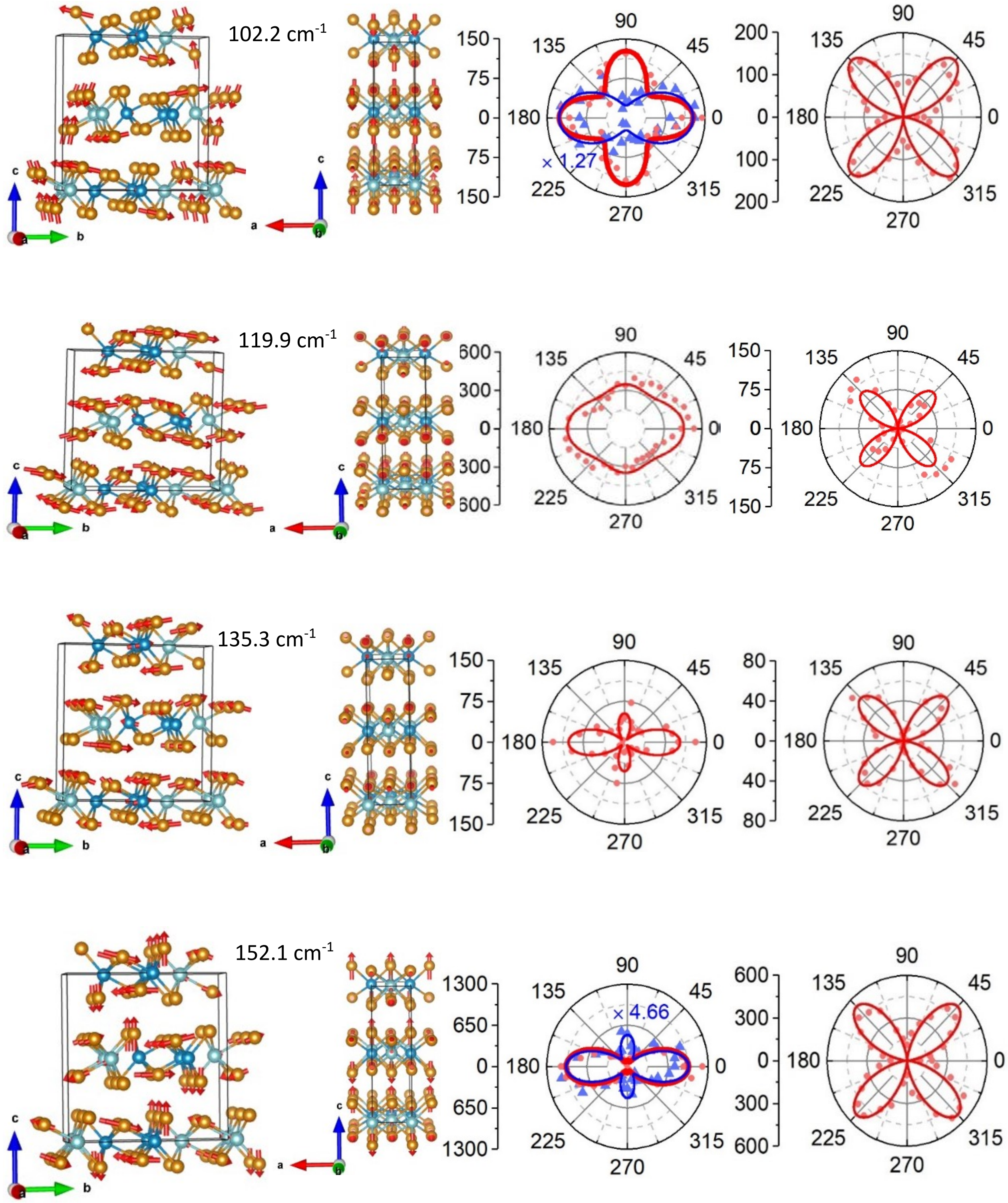

102.2 cm^{-1}
× 1.27
119.9 cm^{-1}
135.3 cm^{-1}
152.1 cm^{-1}
× 4.66
a
b
c
90
45
0
315
270
225
180
135
150
75
200
100
600
300
80
40
1300
650

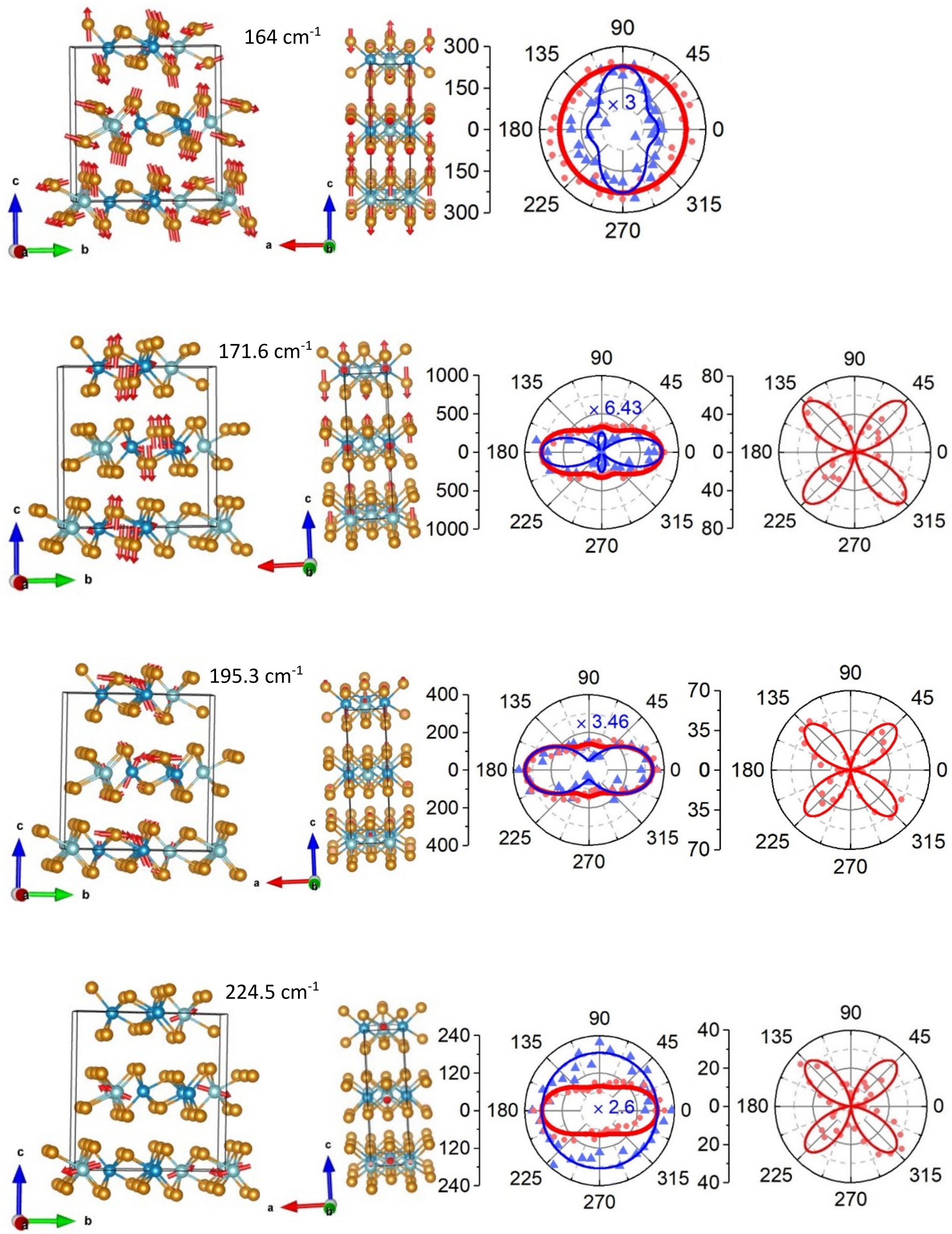

164 cm-1
171.6 cm-1
195.3 cm-1
224.5 cm-1
× 3
× 6.43
× 3.46
× 2.6
a
b
c
90
45
0
315
270
225
180
135

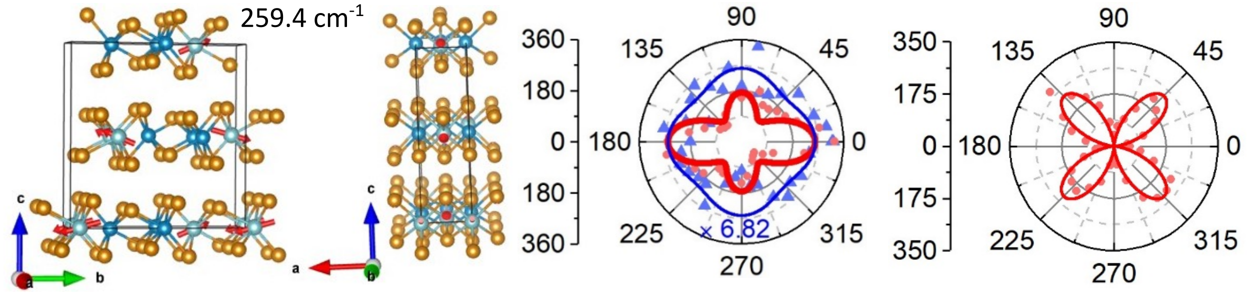

A_2

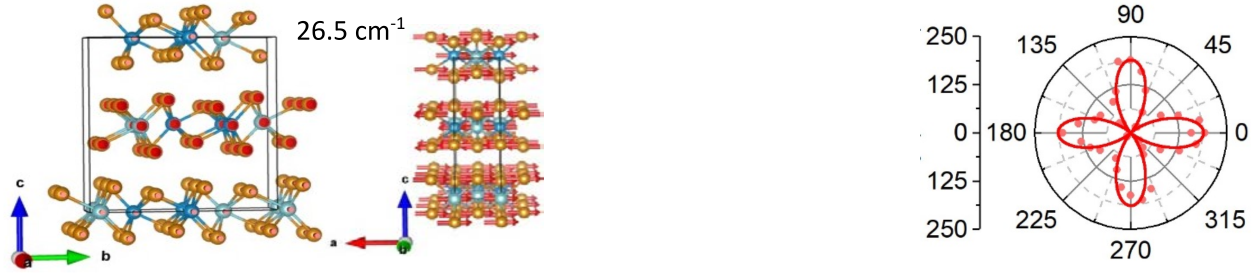

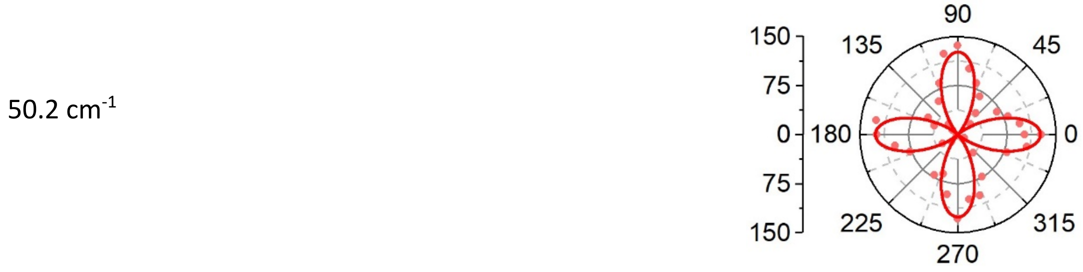

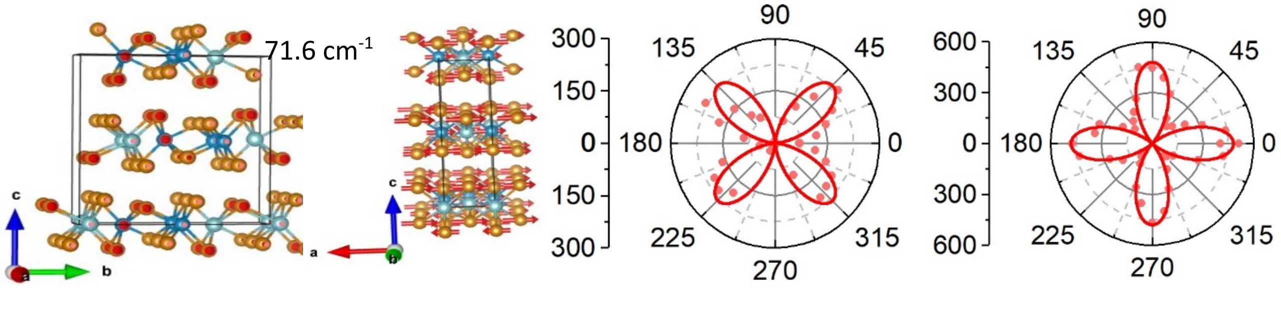

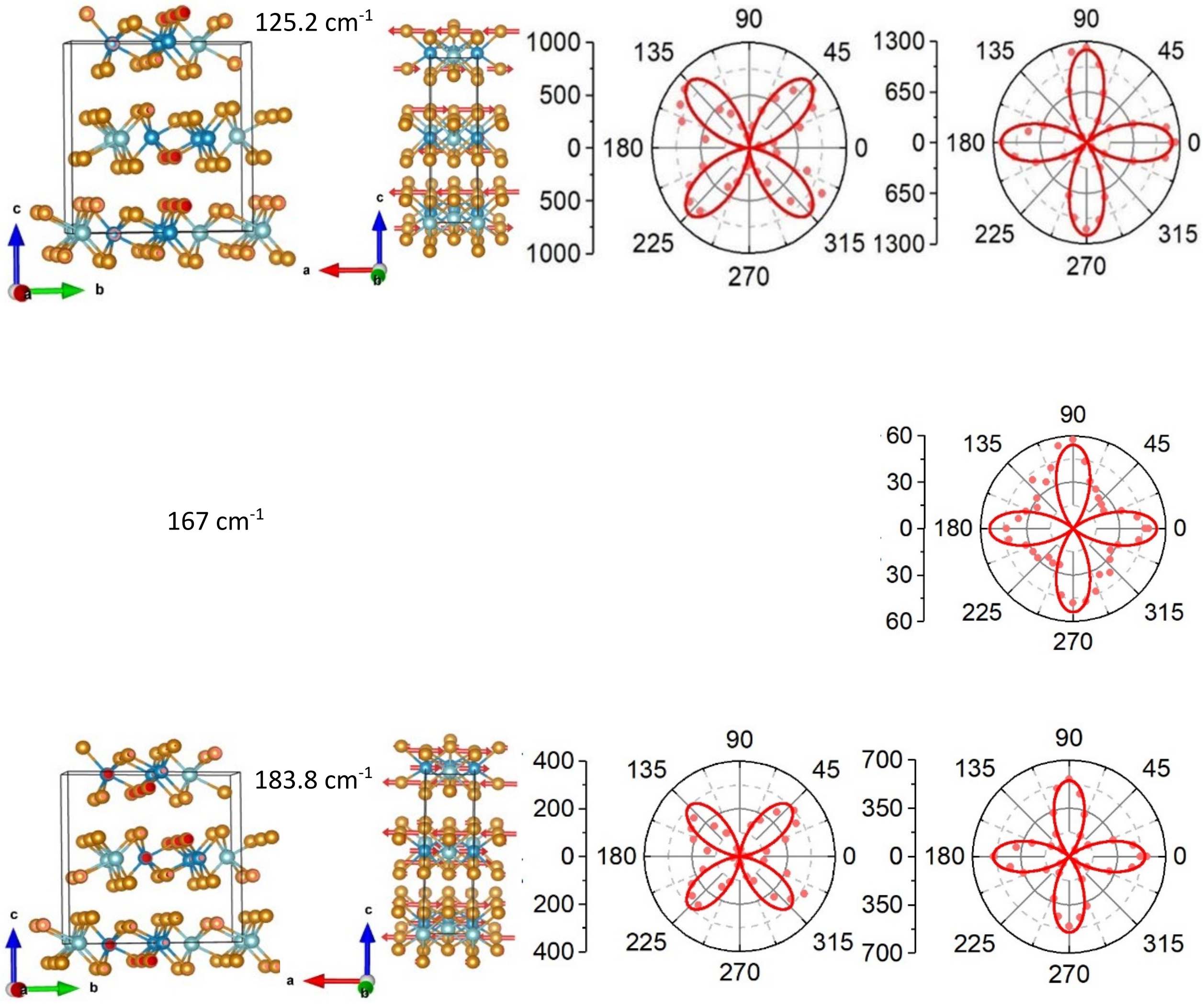

Figure S6: DFT calculation for normal modes of all A_1 and A_2 modes detected in our measurements in two perspective. Second and third columns are parallel and perpendicular measurements, respectively.

S7: Determination of excitation wavelength dependence of Raman tensor elements

We use the *shape* of the angular dependence of the Raman intensity at a particular laser excitation wavelength (either 514 or 633 nm) to determine the quantity |d|/|f| for each A_1 phonon mode. We also measure the change in the Raman intensity for each phonon mode at these two excitation wavelengths as I_{633}/I_{514}. Equation 3 in the main paper can be modified to read:

$$I_{A_1}^{\parallel} = |f|^2\left(\frac{|d|^2}{|f|^2}\cos^4(\theta) + \sin^4(\theta) + 2\frac{|d|}{|f|}\cos^2(\theta)\sin^2(\theta)\cos(\varphi_{df})\right)$$

If the maximum occurs for θ = 0 (this occurs when d < f) then

$$I_{A_1}^{\parallel} = |f|^2\left(\frac{|d|^2}{|f|^2}\cos^4(0)\right)$$

and so one calculates the ratio of the intensities at 633 nm and 514 nm to be

$$\frac{I_{633}}{I_{514}} = \frac{|f_{633}|^2}{|f_{514}|^2}\frac{(|d_{633}|^2/|f_{633}|^2)}{(|d_{514}|^2/|f_{514}|^2)}$$

Thus one obtains the final expression (removing the absolute value symbols for clarity):

$$\frac{|f_{633}|}{|f_{514}|} = \sqrt{\frac{I_{633}}{I_{514}}\frac{|d_{514}|/|f_{514}|}{|d_{633}|/|f_{633}|}}$$

If the maximum occurs for θ = π/2, then

$$I_{A_1}^{\parallel} = |f|^2\left(\sin^4(\pi/2)\right)$$

and so

$$\frac{|f_{633}|}{|f_{514}|} = \sqrt{\frac{I_{633}}{I_{514}}}$$

From this one can directly determine the change in the *d* Raman tensor element as:

$$\frac{|d_{633}|}{|d_{514}|} = \frac{|f_{633}|}{|f_{514}|}\frac{|d_{514}|/|f_{514}|}{|d_{633}|/|f_{633}|}$$

References:

[1] G. Kresse and J. Furthmüller, "Efficient iterative schemes for ab initio total-energy calculations using a plane-wave basis set," *Phys. Rev. B*, vol. 54, no. 16, pp. 11169–11186, Oct. 1996.

[2] G. Kresse and J. Furthmüller, "Efficiency of ab-initio total energy calculations for metals and semiconductors using a plane-wave basis set," *Comput. Mater. Sci.*, vol. 6, no. 1, pp. 15–50, 1996.

[3] G. Kresse and J. Hafner, "Ab initio molecular dynamics for liquid metals," *Phys. Rev. B*, vol. 47, no. 1, pp. 558–561, 1993.

[4] S. Baroni, P. Giannozzi, and A. Testa, "Green's-function approach to linear response in solids," *Phys. Rev. Lett.*, vol. 58, no. 18, pp. 1861–1864, May 1987.

[5] A. Togo, F. Oba, and I. Tanaka, "First-principles calculations of the ferroelastic transition between rutile-type and CaCl2-type SiO2 at high pressures," *Phys. Rev. B*, vol. 78, no. 13, p. 134106, Oct. 2008.
